# Supplementary material for: Targeted two-photon chemical apoptotic ablation of defined cell types in vivo
Source: Nat Commun. 2017 Jun 16;8:15837. doi: 10.1038/ncomms15837 (PMC5501159; doi:10.1038/ncomms15837)
Supplement: Supplementary Information — Supplementary Figures [file ncomms15837-s1.pdf]

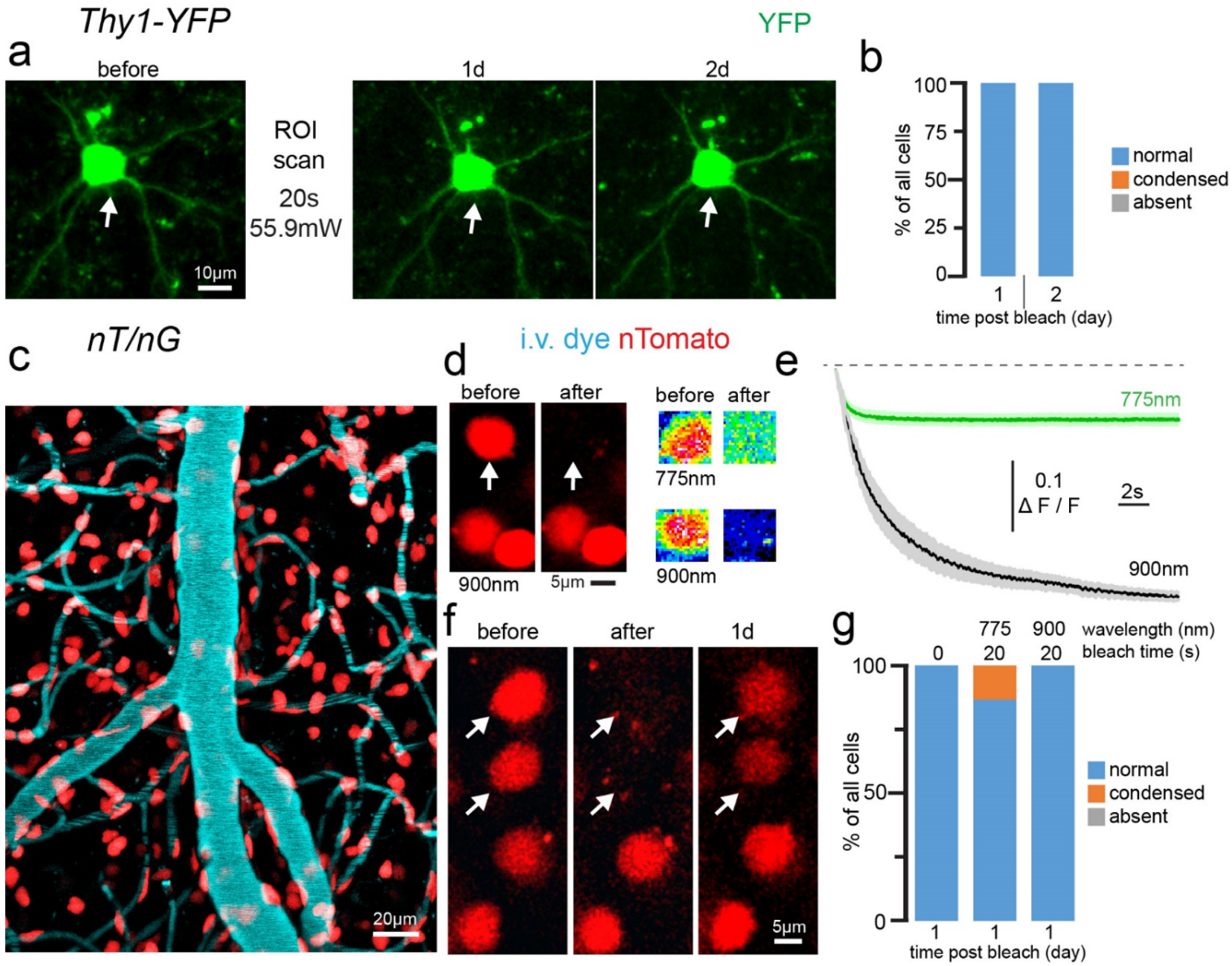

Supplementary Figure 1: Cytoplasmic and nuclear localized fluorescent protein photo-bleaching is not sufficient to reliably induce apoptosis

(a) In vivo images of a YFP labeled neuron (arrow) that underwent ROI based 775nm laser scanning for 20 seconds at 55.9 mW laser intensity showing no induction of cell death over the following days. **(b)** Quantification showing the lack of cell death after YFP only photo-bleaching for 20 seconds at (33-55mW, 775nm) laser intensities which were sufficient to induce cell death with H33342 but not with YFP only, n=18 cells from 3 mice. **(c)** In vivo image of nuclear localized Tomato (nTomato) expression in nT/nG transgenic mice. **(d)** Robust photo-bleaching of nTomato at both 775nm and 900nm laser wavelengths. **(e)** Fluorescence intensity traces showing photo-bleaching of nT/nG at the laser intensities indicated, traces indicate mean ± SEM **(f)** In vivo time lapse images showing two cells targeted for photo-bleaching (arrows) with no evidence of nuclear condensation or disappearance the following day. **(g)** Quantification of induced cell death after nTomato photo-bleaching for 20 seconds at high laser intensity at both 775nm and 900nm n=15 cells per wavelength.

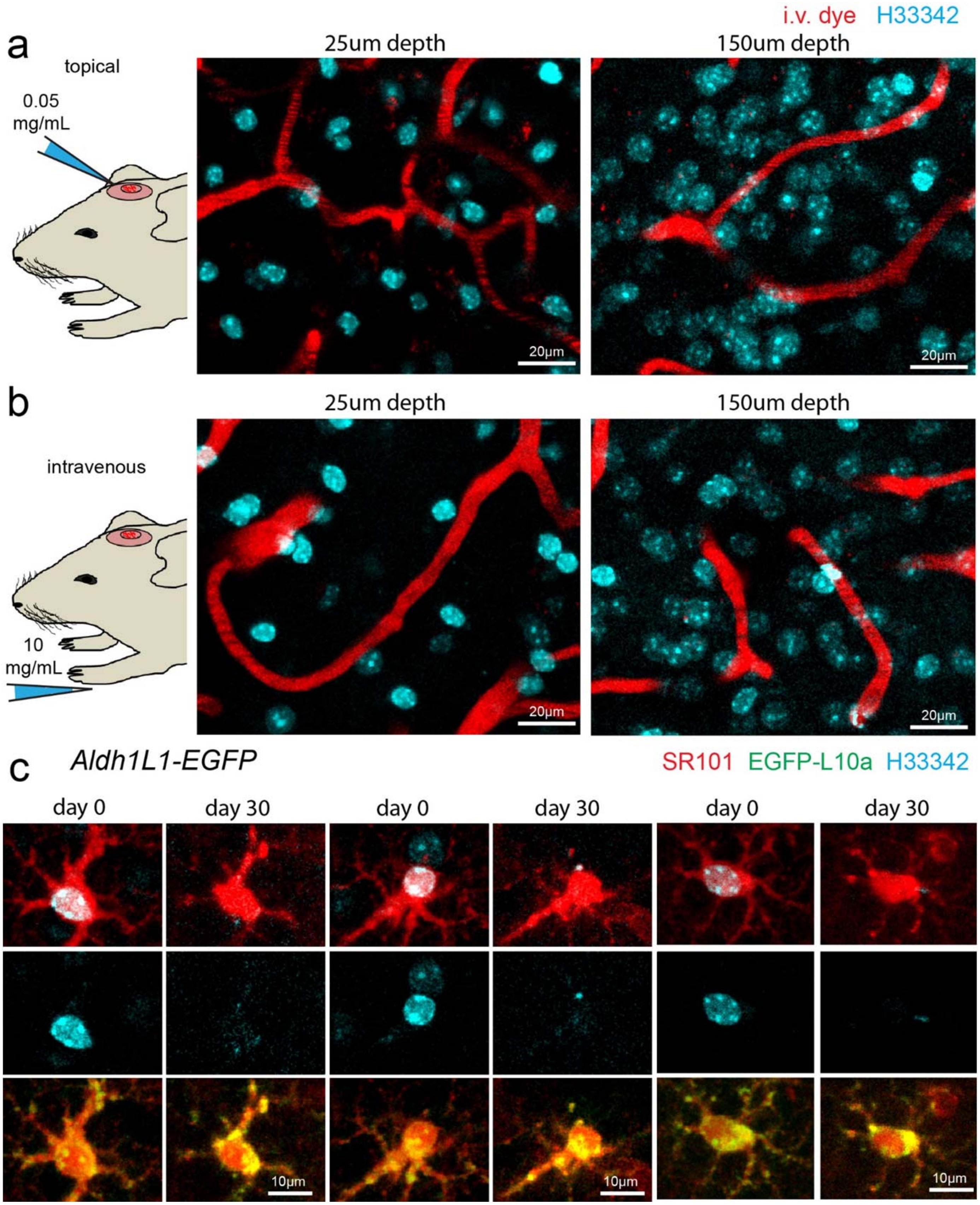

Supplementary Figure 2: In vivo H33342 labeling and dye removal over time

(a-b) Schematic and representative in vivo images showing H33342 labeling after topical or intravenous dye injection at the indicate depths. **(c)** In vivo time lapse imaging showing the loss of H33342 in single astrocytes identified by SR101 and Aldh1L1-EGFP labeling imaged 30 days apart.

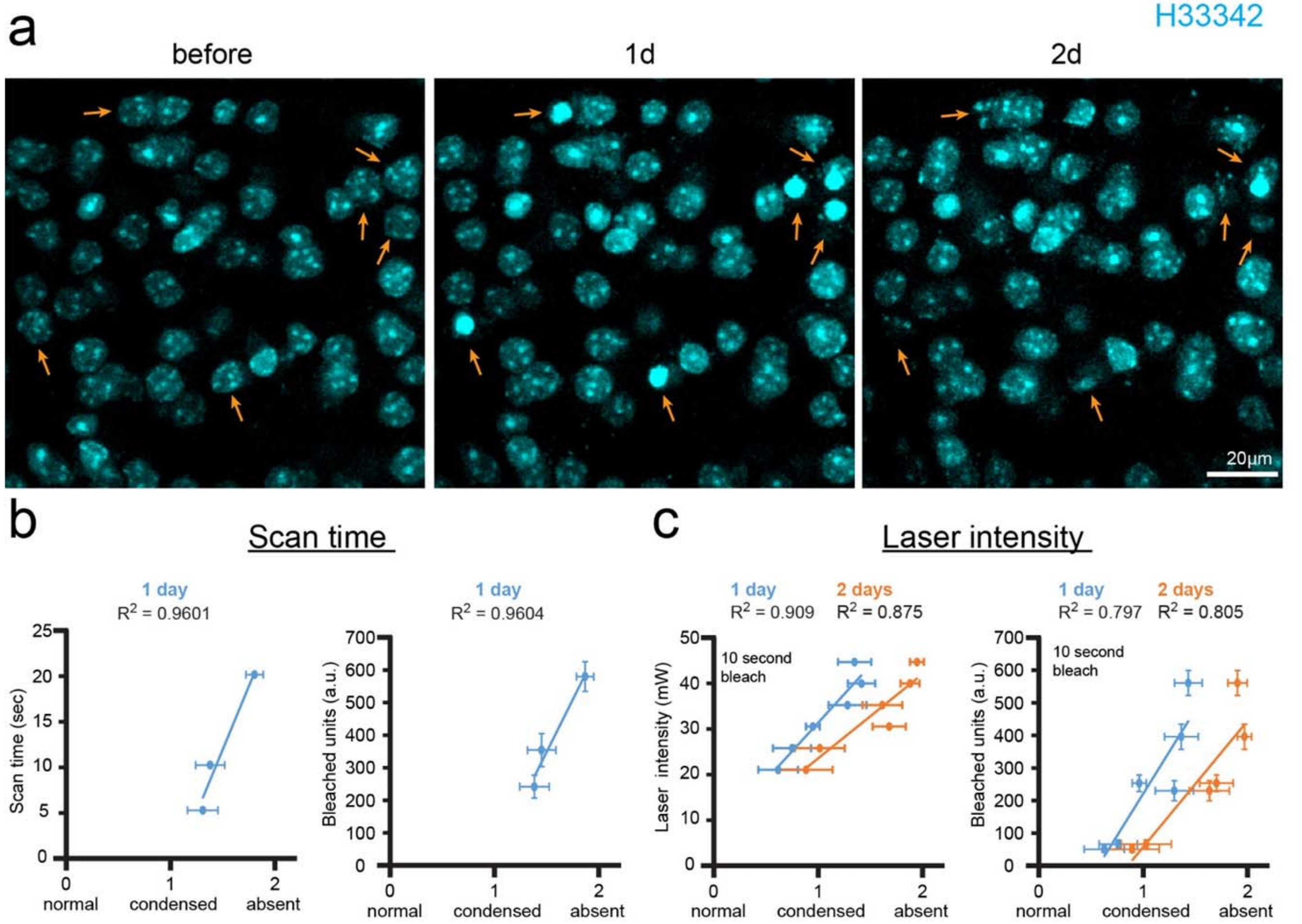

Supplementary Figure 3: Scan time and laser intensity dose-dependent induction of apoptosis in vivo
(a) In vivo time lapse images demonstrating *2Phatal* induced condensation and disappearance of targeted cells (arrows). **(b)** Linear correlations between scan time and detection of cell death and units bleached per cell and cell death, traces indicate mean ± SEM. **(c)** Linear correlations between laser intensity and detection of cell death and units bleached per cell and cell death, traces indicate mean ± SEM

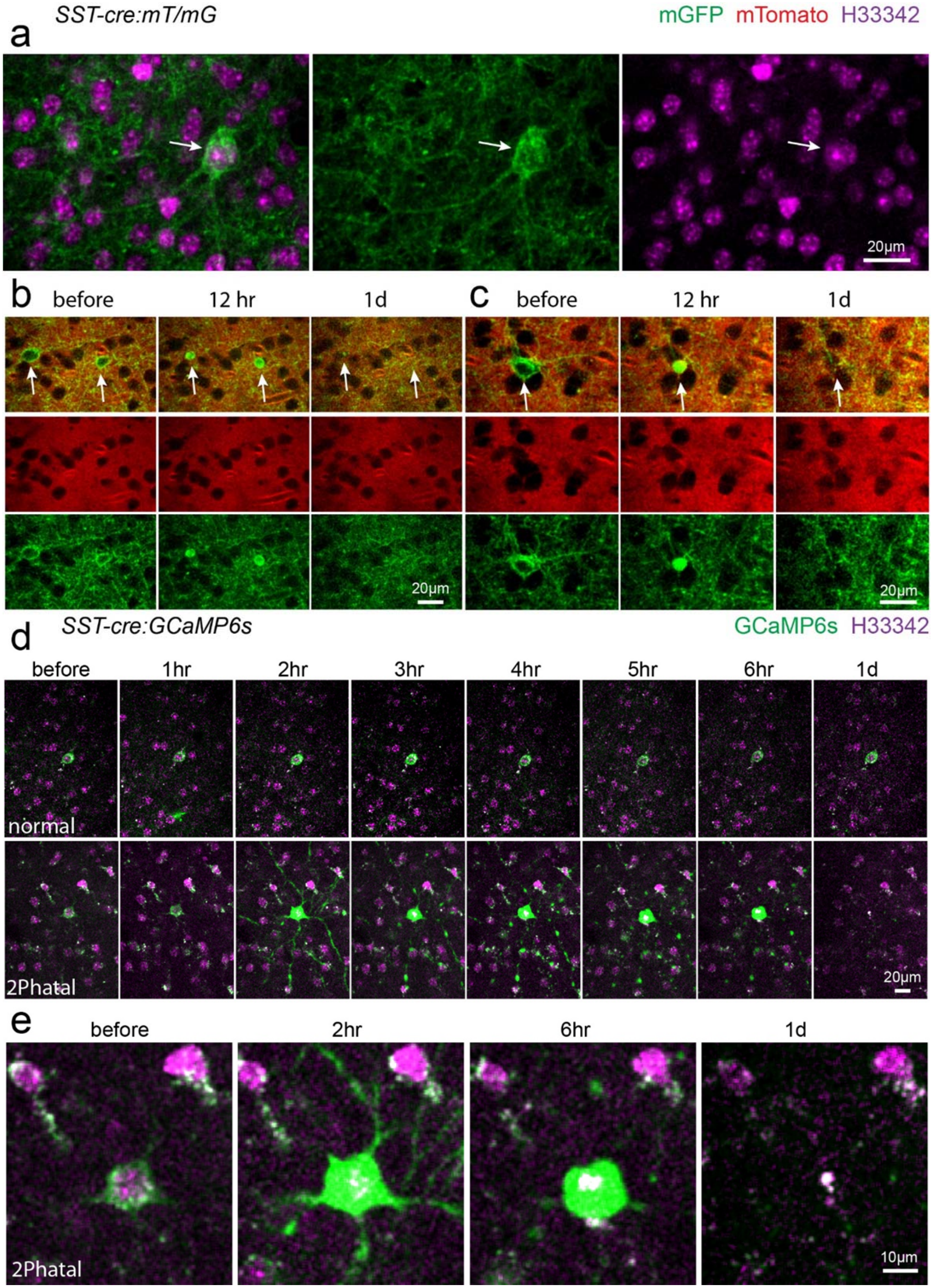

Supplementary Figure 4: 2Phatal of somatostatin interneurons

(a-c) In vivo images of membrane bound GFP in SSTcre:mT/mG mice showing *2Phatal* induction of apoptosis in targeted somatostatin interneurons (arrows). **(d-e)** In vivo time-lapse sequences of control or *2Phatal* somatostatin interneurons with GCaMP6s expression showing distinct calcium overload in the targeted cell soma and dendrites 2-6 hours after *2Phatal* photo-bleaching.

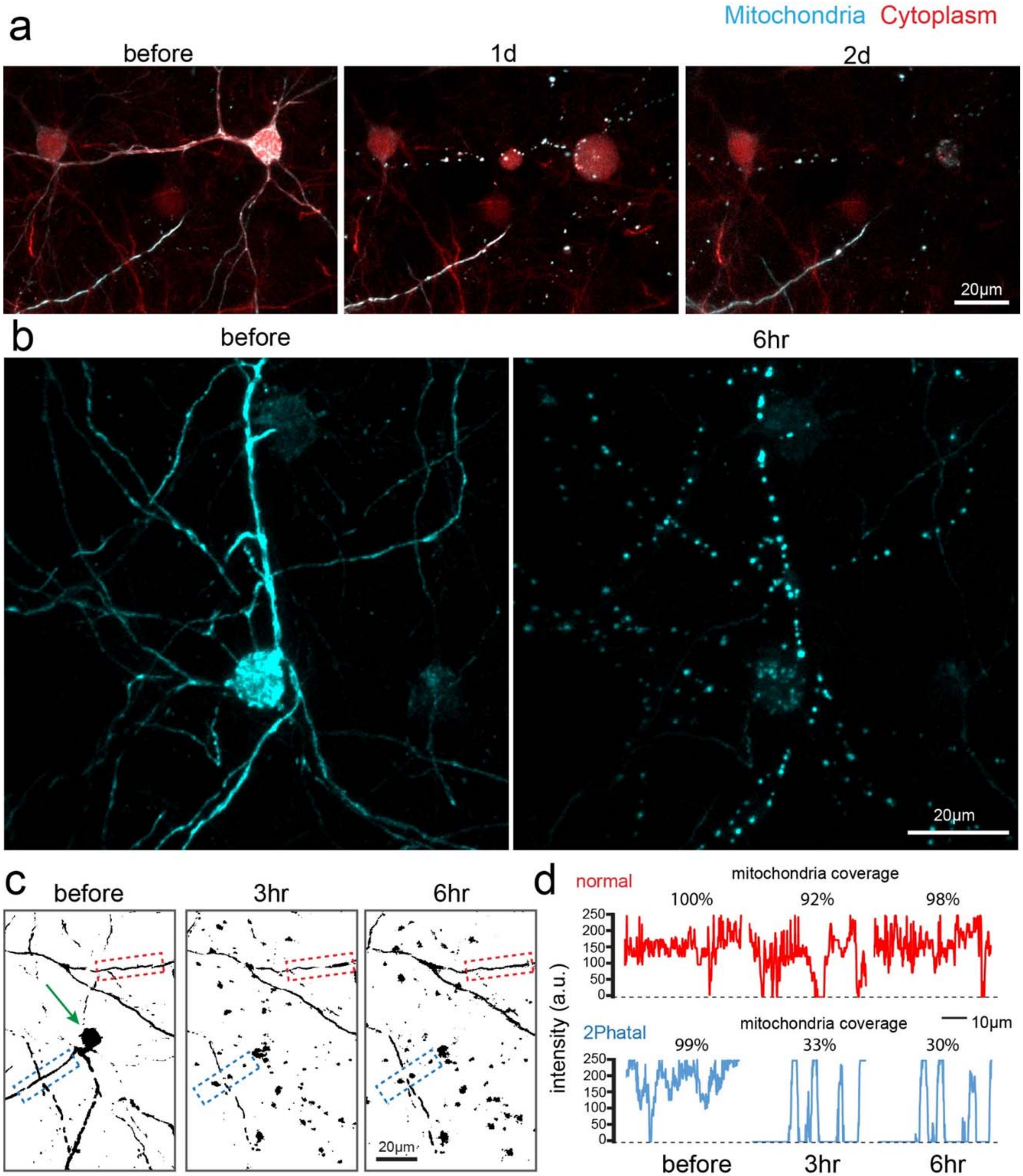

Supplementary Figure 5: Mitochondrial fission during 2Phatal of neurons

(a) In vivo time lapse imaging of mitochondrial labeling in a single *2Phatal* targeted neuron showing mitochondrial fission during apoptosis. **(b)** In vivo time lapse imaging showing mitochondrial fission in a single neuron targeted for *2Phatal*. **(c-d)** Representative images and quantification showing thresholding of the mitochondrial signal and changes in the signal and mitochondrial coverage at 3 and 6hrs during the mitochondrial fission. Arrow indicates neuronal cell body and boxed regions in **(c)** correspond to the traces shown in **(d)**.

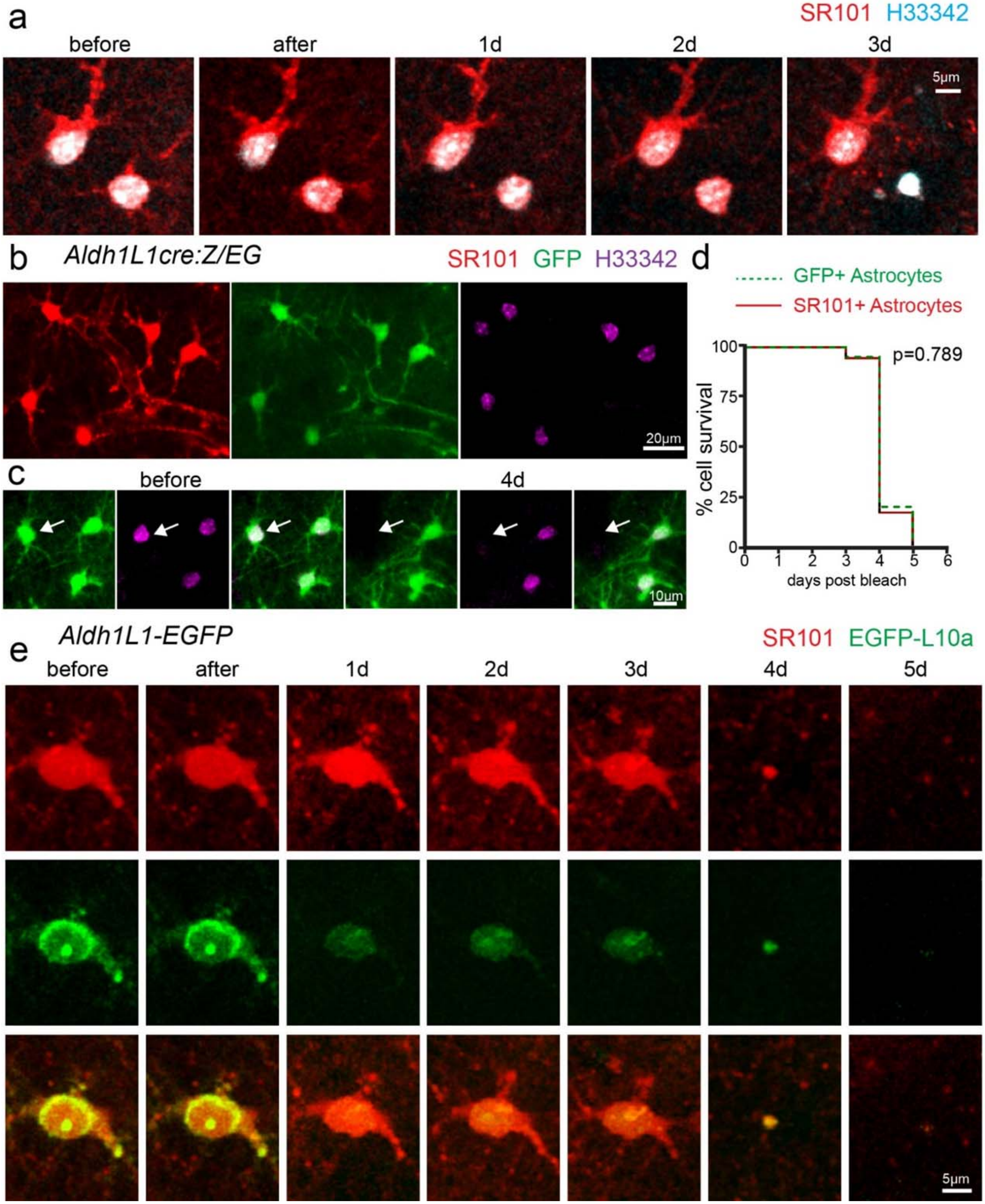

Supplementary Figure 6: 2Phatal of astrocytes and early ribosomal disassembly

(a) In vivo time-lapse sequence showing apoptosis initiation characterized by nuclear pyknosis and formation of apoptotic bodies on day 3 after *2Phatal* photo-bleaching. **(b-c)** In vivo images of H33342 labeling in an Aldh1L1cre:Z/EG transgenic mouse showing ablation of a single cell (arrows). **(d)** Comparison between GFP only labeled astrocytes and SR101 labeled astrocyte cell survival after *2Phatal* photo-bleaching showing no significant difference in apoptosis initiation (GFP: n= 41 cells from 3 mice; SR101: n=18 cells, from 4 mice) Log-rank (Mantel-Cox) test: Chi square 0.07144, degrees of freedom=1. **(e)** In vivo time lapse sequence showing early disassembly and loss of the EGFP-L10a ribosomal tagged GFP in a single astrocyte targeted with *2Phatal*.

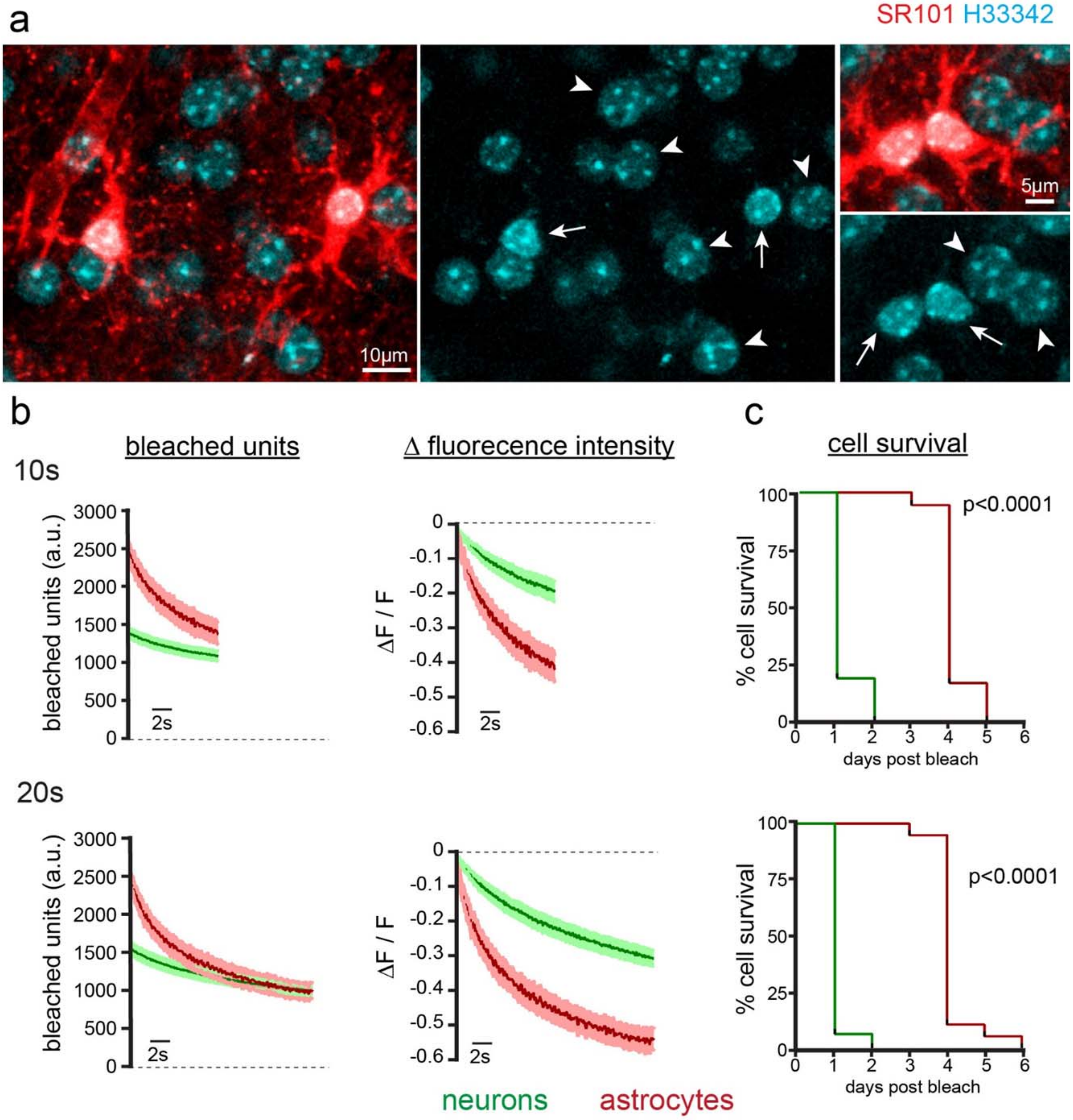

Supplementary Figure 7: Differential apoptosis initiation between neurons and astrocytes.

(a) In vivo images showing brighter H33342 labeling of SR101-labeled astrocytes (arrows) compared to adjacent neurons (arrowheads). **(b)** Quantification comparing the total bleached units per cell (left) and overall change in fluorescence intensity compared to baseline (right) during 10 and 20 second *2Phatal* photo-bleaching in both neurons and astrocytes. Astrocytes are initially brighter (Figure 1c) and thus bleach more, traces indicate mean ± SEM. **(c)** Comparison between neuron and astrocyte cell survival after *2Phatal* photo-bleaching showing significantly delated apoptosis initiation in astrocytes (n=18 cells, 10 s scan; and 19 cells, 20s scan; from 4 mice) compared to neurons (n=16 cells, 10s scan; and 16 cells, 20s scan, from 4 mice), Log-rank (Mantel-Cox) test : 10s-Chi square 33.78, degrees of freedom=1; 20s-Chi square 36.96,degrees of freedom=1.

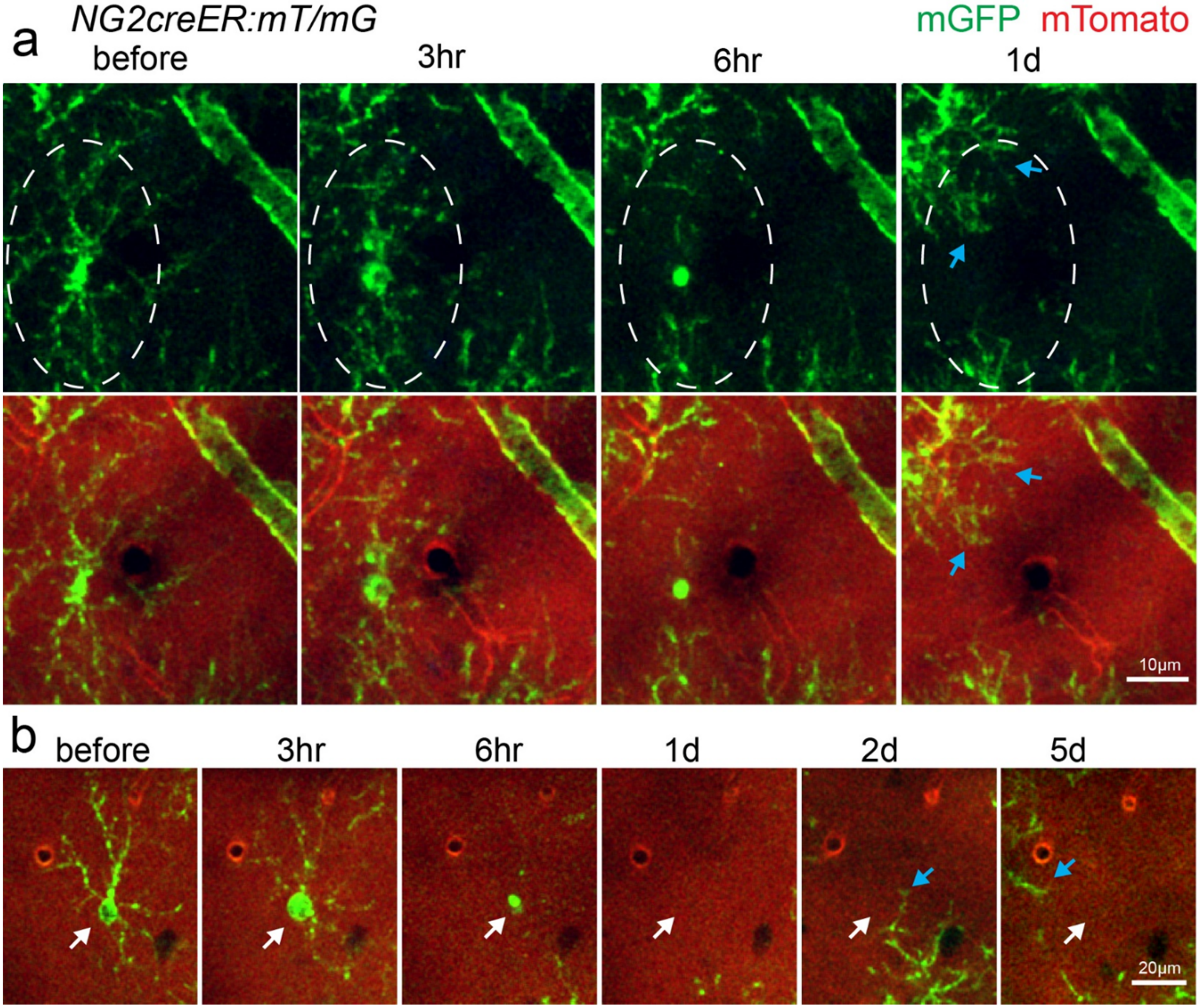

Supplementary Figure 8: NG2 glia ablation and territory invasion by neighboring cells
(a-b) In vivo time lapse sequences showing apoptosis of single NG2 glia (white arrows) with territory invasion by neighboring cells over the following days (blue arrows).
